# Comparison of Surgical Outcomes Associated With Compression Secondary to Hemorrhage and Intervertebral Disk Extrusions in Dogs

**DOI:** 10.3389/fvets.2022.889113

**Published:** 2022-07-04

**Authors:** Patricia E. Lawler, Jonathan H. Wood, Nicole E. Alleva, Mark Rishniw, Ian Porter, Phillipa J. Johnson

**Affiliations:** ^1^Department of Clinical Sciences, Cornell University School of Veterinary Medicine, Ithaca, NY, United States; ^2^Department of Small Animal Medicine and Surgery, College of Veterinary Medicine, University of Georgia, Athens, GA, United States

**Keywords:** intervertebral disk, magnetic resonance imaging, epidural hemorrhage, hemilaminectomy, dog

## Abstract

Acute intervertebral disk extrusion (IVDE) is one of the most commonly reported neurologic disorders seen in veterinary practice. There is a recognized subset of IVDE cases that have a hemorrhagic inflammatory reaction within the epidural space that causes compression in addition to compression from herniated disk material. Previous reports have been conflicting in the outcomes of these cases. The goals of this retrospective case-control cross-sectional study are to (1) compare the success rate of routine surgical decompression in dogs with DEEH compression compared to Modified Frankel Score (MFS) matched dogs with non-hemorrhagic disk extrusions; (2) evaluate the extent of spinal cord compression on MRI compared to final patient outcomes in DEEH compression and (3) determine the surgical compression to decompression ratio and its relation to patient outcomes in cases of DEEH compression. A total of 143 dogs were included in this study and divided into two groups: DEEH compression dogs (*n* = 78) and non-hemorrhagic IVDE dogs (*n* = 65). Outcomes were assigned for each patient [0 = deceased, 1 = alive and non-ambulatory (MFS 0–3), 2 = alive and ambulatory (MFS 4 or 5)] in both groups. Outcomes of DEEH and non-hemorrhagic IVDE did not differ when taken to surgery with comparable success rates when stratified by MFS. Similarly, outcomes did not differ between DEEH and non-hemorrhagic IVDE dogs when assessed by compression to decompression ratio. Dogs with DEEH compression had more compressed sites than dogs with non-hemorrhagic IVDE (*P* = 0.001) and had more sites decompressed surgically than dogs with non-hemorrhagic IVDE (*P* < 0.001). Consequently, the compression to decompression ratio did not differ between the two groups (*P* = 0.52). Our results support the finding that when a similar level of surgical decompression is achieved, dogs with DEEH compression have similar outcomes to dogs with non-hemorrhagic IVDE for similar degrees of neurological dysfunction.

## Introduction

The incidence of intervertebral disk extrusion (IVDE) in dogs is approximately 2%, although chondrodystrophic breeds, such as Dachshunds and Beagles, have a much greater risk of developing IVDE. Furthermore, ~4% of veterinary emergency room visits are due to acute severe thoracolumbar spinal cord injuries, 74% of which are due to IVDE (1, 2). Few studies report the presence of hemorrhage associated with IVDE, and its incidence is unknown (3). Some investigators have termed intervertebral disk disease with hemorrhage “disk extrusions with extensive epidural hemorrhage” (DEEH) (4, 5).

Despite this naming convention the histopathological changes of this compressive material have consistently been shown to include more than just hemorrhage (5–8). With DEEH compression, hemorrhagic inflammation can spread several levels cranial and caudal to the original site of herniation, resulting in additional extradural compression of the spinal cord (5, 9). Inflammation secondary to the presence of disk material and hemorrhage in the spinal canal can lead to acute clinical signs of a myelopathy.

Previous investigators have provided differing prognoses for dogs with DEEH compared to dogs with non-hemorrhagic IVDE. Tartarelli et al. described that the recovery rate for a case series of 23 dogs with DEEH compression was ~91% (9). Mateo et al. also found similar outcomes (93–95%) in 46 dogs with DEEH compression compared to 50 unmatched control dogs with non-hemorrhagic IVDE (8). In contrast, Woelfel et al. in a case series of 59 medium-to-large-breed dogs with DEEH compression, observed that only 77.3% of paraplegic dogs, and 37.8% of dogs with absent pain perception, regained ambulation (4). This study found an association between larger surgical decompressions and improved outcomes in their subpopulation of dogs. This study did not evaluate the extensive length of compressive material on T2W images in relation to the length of surgical decompression.

Therefore, we sought to examine whether the amount of compression differed between dogs with DEEH and those with non-hemorrhagic IVDE. Further, we examined whether the relative amount of decompression would affect outcomes in these two groups of dogs. We hypothesized that dogs with DEEH compression would have more sites of compression on MRI, would require more decompressive surgery to relieve this compression, and would have similar outcomes for the same compression to decompression ratio as dogs with IVDE without hemorrhagic inflammation.

## Materials and Methods

Medical records of dogs presented to the Cornell University Hospital for Animals (Ithaca, NY) between January 2010 and January 2020 were reviewed. Cases were included if the following criteria were fulfilled: (2) dogs had clinical signs of thoracolumbar myelopathy, (3) dogs underwent a thoracolumbar MRI, (5) intervertebral disk herniation was diagnosed on the finalized radiology report, (8) dogs underwent routine hemilaminectomies. Cases were excluded if medical records were incomplete, if dogs were euthanized prior to surgery or if dogs were lost to follow-up. The cases were divided into two groups- a DEEH compression group where IVDE and hemorrhagic extradural compression were included in the final radiology report and a control group representing non-hemorrhagic intervertebral disk disease.

Data were collected from medical records and included signalment (age, sex, weight, breed), presenting clinical signs, location of compression on MRI, surgical procedure, side and site of surgery, and length of time in hospital. A Modified Frankel Score (0 = paraplegic deep pain negative, 1 = paraplegic deep pain positive, 2 = paraplegic superficial pain intact, 3 = non-ambulatory paraparesis, 4 = thoracolumbar pain only, 5 = normal patient) was assigned to each case based on the medical records at presentation, discharge, and follow-up examination. Cases with incomplete medical records were excluded from the study.

After the total number of cases in the DEEH compression was assembled, a control group of non-hemorrhagic IVDE dogs was assembled. These were cases with an MRI and surgery report available for review and over the same time frame as the original search. They were organized into a spreadsheet based on Modified Frankel Score at admission, and cases were randomly selected for inclusion until the case count for MFS in each group was the same. No other patient criteria were used for matching patients. Follow-up phone calls were made if the pet had not returned for a recheck examination by the time of the study and included questions regarding patient outcome and whether the pet returned to ambulatory status. Based on the follow-up Modified Frankel Score, the cases were assigned an outcome score [0 = deceased, 1 = alive and non-ambulatory (MFS 0–3), 2 = alive and ambulatory (MFS 4–5)].

The following MRI sequences were available for review and comparison in all cases: T1- and T2- weighted spin echo sagittal and transverse images. Additional sequences that were reviewed when available included STIR, T1w fat-saturated, T2^*^ (T2 weighted gradient echo or “susceptibility-weighted”), and post-contrast (gadolinium IV) T1 weighted sagittal and transverse images. Each MRI was reviewed by a board-certified neurologist (ACVIM) and neurology resident for inclusion in this study.

MRIs were assigned to the control group if the extradural material attenuated more than 20% of the cross-sectional area of the vertebral canal on a T2W transverse image and had no hemorrhagic compressive material extending beyond 1 vertebral body cranial or caudal to the site of extrusion on T2w sagittal images. MRIs were assigned to the DEEH compression group if the extradural material attenuated more than 20% of the cross-sectional area of the spinal canal at sites more than 1 vertebral body away from the site of extrusion. This material could be T2w hyper-, iso-, or hypo-intense. Where T2^*^ was available, the material was assumed to contain hemorrhage if the material demonstrated signal void.

**Figure 1:**
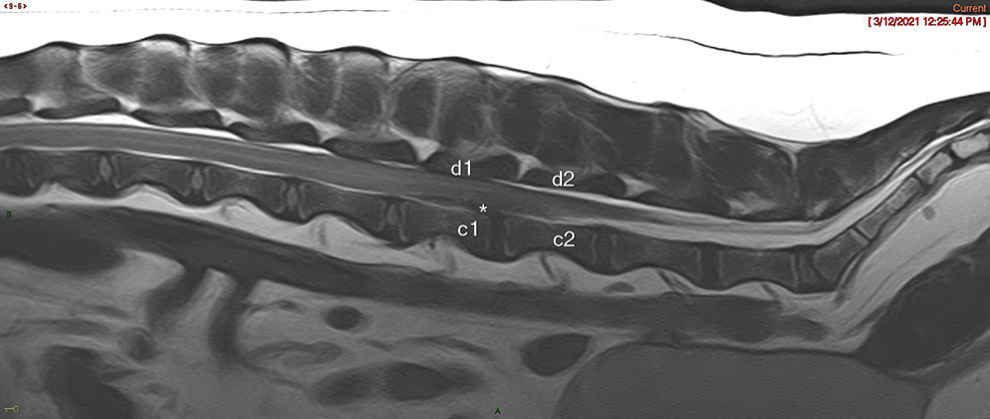
T2w Sagittal MRI image showing spinal compression count noted as “c” and surgical decompression count noted as “d.” This patient had a compression:decompression ratio of 1. An * denotes representative areas of intervertebral disk herniation.

**Figure 2:**
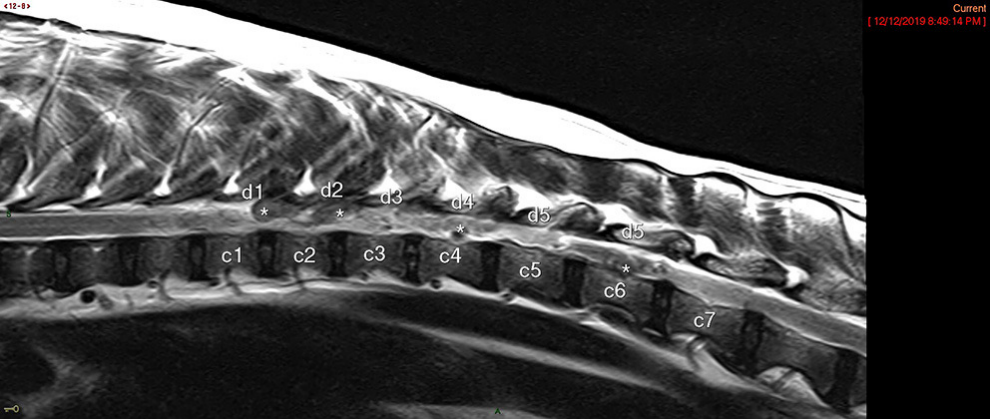
T2w Sagittal MRI image showing spinal compression count noted as “c” and surgical decompression count noted as “d.” This patient had a compression:decompression ratio of 1.16. An * denotes representative areas of DEEH intervertebral disk herniation.

The number of vertebral laminae overlaying >a 20% attenuation of the vertebral canal was calculated for each case based on review of the imaging and finalized radiology reports. For the DEEH compression population, a T2W vertebral length ratio was calculated for each case based on the T2w sagittal image as previously described (10). A compression to surgical decompression ratio was calculated by dividing the number of laminae over which the spinal cord was compressed by the number of laminectomies performed (Figure 1). Therefore, a ratio of <1 indicates a case where more laminae were surgically decompressed than laminae overlying compression were noted on MRI and vice versa (Figure 2).

### Statistics

Age, number of compressed sites, number of sites surgically decompressed, the compression to decompression ratio, Modified Frankel Score at admission and follow-up, and the years during which cases were seen were compared using Rank Sum tests. The outcomes (success vs. failure) between the groups were first compared using a Mantel-Haenszel test to examine whether differences existed between the two groups after controlling for the Modified Frankel Score at admission. If different Modified Frankel Scores did not affect the outcome between groups, we would then use a chi-square test with a Yates continuity correction after combining all levels of severity in each group. Finally, we looked at the effect of the level of severity on the probability of a successful outcome with a chi-square test with a Yates continuity correction. We excluded dogs euthanized prior to discharge (2 dogs in each group).

## Results

We identified 658 dogs diagnosed with IVDE; of these, 169 dogs met inclusion criteria: 91 dogs with evidence of DEEH compression and 78 dogs with non-hemorrhagic IVDE that were randomly selected to match the modified Frankel scores of the DEEH compression group. DEEH cases compromised 13.8% of IVDE cases in our hospital from 2010 to 2020. Of the 91 dogs with DEEH compression, 17 were euthanized after MRI, but prior to surgery, 6 dogs were lost to follow-up, and 3 dogs underwent dorsal laminectomies—these 26 dogs were excluded from analysis. Therefore, we retained 65 dogs with DEEH compression and 78 dogs with non-hemorrhagic IVDE.

### Control Group

At diagnosis, dogs had a median age of 6.14 years (range 2–14 years) and mean body weight of 11.9 kg (range 4–40.7 kg); 33 dogs were spayed females, 40 were castrated males, 3 were intact males, and 1 was an intact female. The dog breeds represented included mixed breed (34), Miniature Dachshund (20), French bulldogs (10), Beagle (5), German Shepherd Dog (3), Staffordshire Terrier (3), and one of each—Cardigan Welsh Corgi, Pembroke Corgi, Bolognese, Shih Tzu, Havanese, Miniature Poodle, Cocker Spaniel, and Pug. Based on intake neurologic examination, lesions in 72 dogs localized to the T3–L3 region, one localized to L4–S3, and 5 had multifocal (T3–L3 and L4–S3) lesions.

All dogs underwent routine hemilaminectomy by a training neurology or surgery resident under the supervision of a board-certified neurologist.

### DEEH Compression Group

**Figure 3:**
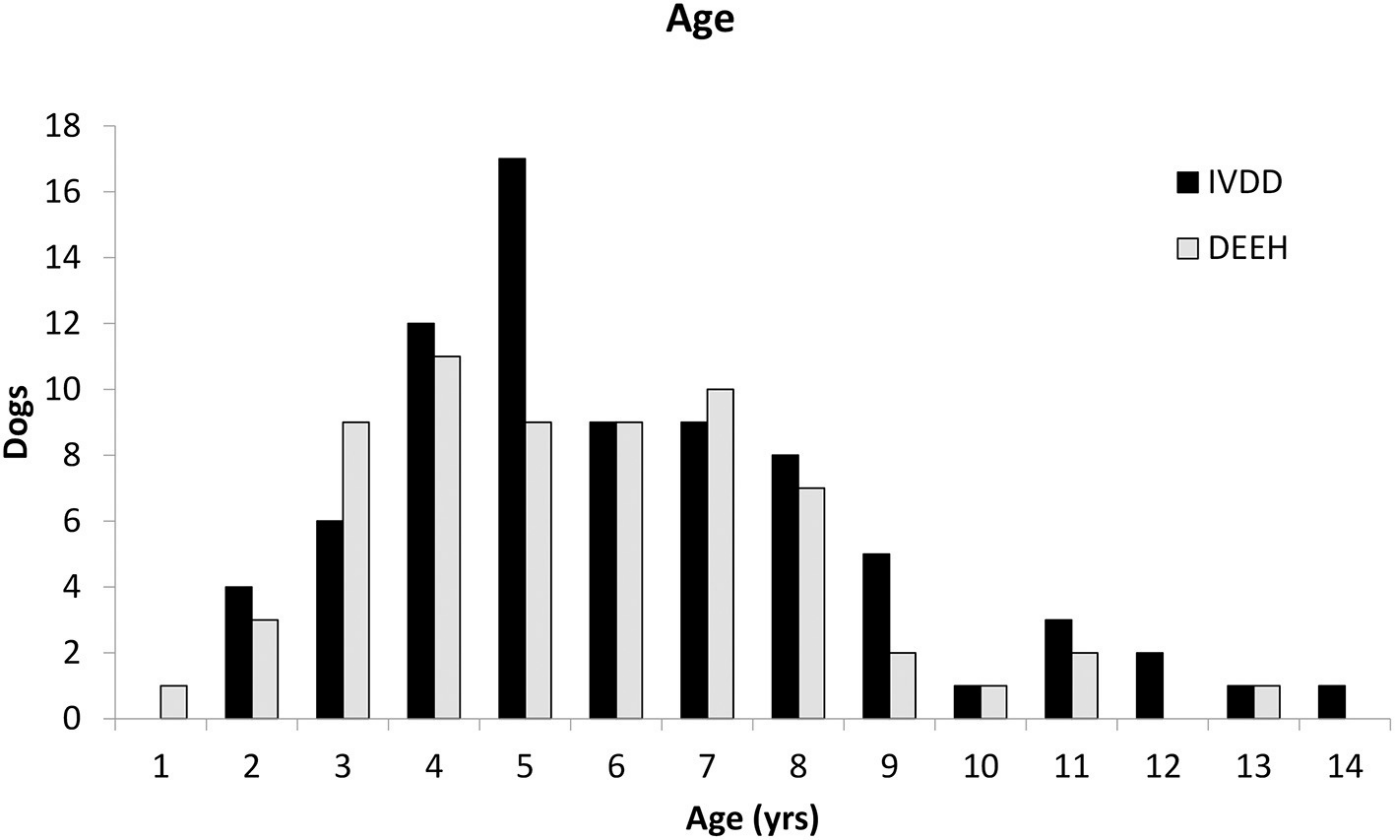
Age distribution of patients with IVDE and DEEH.

**Figure 4:**
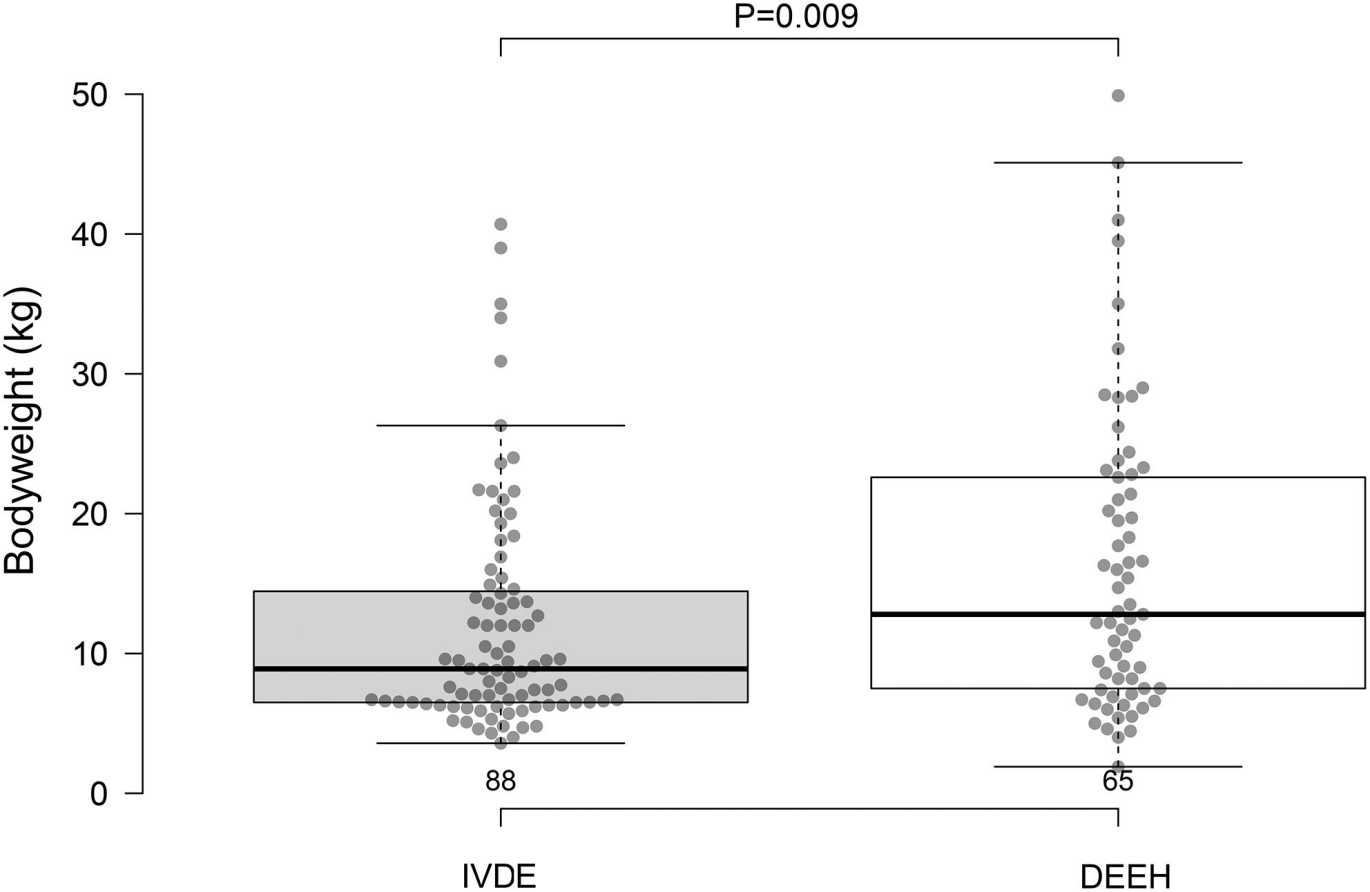
Body weights (kg) for IVDE control group and DEEH compression group.

At diagnosis, dogs had a median age of 5 years (range 2–13 years), which did not differ from the control dogs (*P* = 0.322; Figure 3). The mean body weight was 16.1 kg (range 1.9–53.1 kg) which was significantly larger than those of the control group (*P* = 0.009; Figure 4). Thirty dogs were spayed females, 29 were castrated males, 3 were intact males, and 3 were intact females. The dog breeds represented included mixed breed (26), Miniature Dachshund (11), French Bulldog (10), German Shepherd Dog (10), Beagle (10), Border Collie (3), Husky (3), Cocker Spaniel (3), and one of each—Bolognese, Cardigan Welsh Corgi, Coton de Tulear, Labrador Retriever, Shetland Sheepdog, Shih Tzu, Staffordshire Bull Terrier, and Whippet. Based on intake neurologic exam, lesions in 50 dogs localized to the T3–L3 region, 5 localized to L4–S3, and 10 had multifocal (T3–L3 and L4–S3) lesions.

All dogs underwent routine hemilaminectomy by a training neurology or surgery resident under the supervision of a board-certified neurologist.

### Compression Sites and Decompression Extent

**Figure 5:**
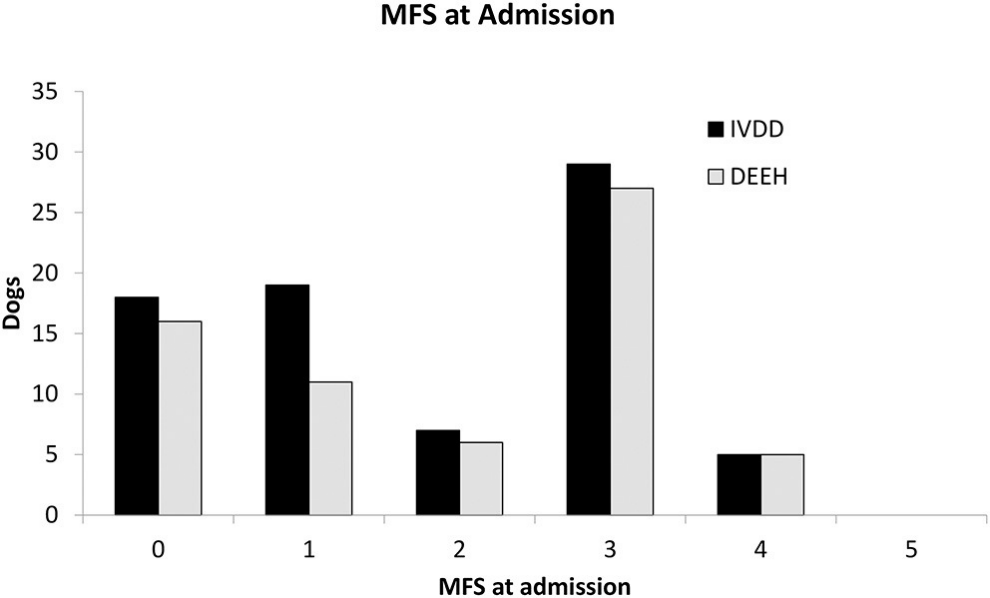
Modified Frankel Score (MFS) at the time of admission for cases with IVDE and DEEH.

**Figure 6:**
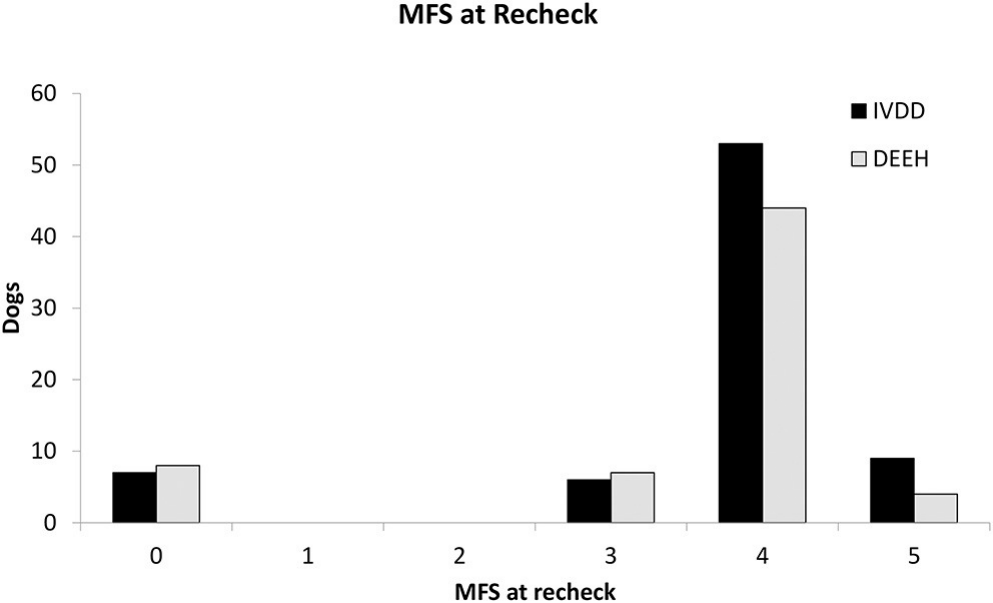
Modified Frankel Score (MFS) at the time of follow-up for cases with IVDE and DEEH.

Dogs with DEEH compression had more compressed sites (median: 4; range 2–12) than dogs with non-hemorrhagic IVDE (median: 2; range 2–7; *P* = 0.001). Dogs with DEEH compression had more sites decompressed surgically (median: 4; range 2–9) than dogs with non-hemorrhagic IVDE (median: 2; range 2–6; *P* < 0.001). The compression to decompression ratio did not differ between the two groups (*P* = 0.52). Modified Frankel Scores at admission (Figure 5) and recheck (Figure 6) did not differ between the two groups (*P* = 0.65 and 0.15, respectively).

### Outcomes

Follow-up examinations for dogs with IVDE occurred at a median of 3 weeks post-operatively (range 3–50 weeks). At the time of follow-up, sixty-two dogs (79%) regained ambulation; 13 dogs (17%) were alive but had not regained ambulation, and three dogs (4%) were euthanized in the initial postoperative period due to clinical signs consistent with ascending myelomalacia (Table 1).

**Table 1:** Modified Frankel Score distribution and successful outcome.

	**Admit number of cases**		**Successful outcome number of cases (%)**	
**MFS**	**DEEH compression**	**Control**	**DEEH compression**	**Control**
0	16	19	4 (25)	6 (32)
1	11	19	9 (82)	17 (90)
2	6	7	4 (67)	7 (100)
3	27	28	25 (93)	27 (96)
4	5	5	5 (100)	5 (100)
Total	65	78	47 (72)	62 (79)

Follow-up examinations for dogs with DEEH compression occurred at a median of 6 weeks post-operatively (range 3–200 weeks). At the time of follow-up, forty-seven dogs (72%) regained ambulation; 14 dogs (22%) were alive but did not regain ambulation, 2 dogs (3%) survived to discharge but were euthanized due to continued clinical signs, and two dogs (3.0%) were euthanized in the immediate postoperative period while hospitalized due to clinical signs consistent with ascending myelomalacia.

**Figure 7:**
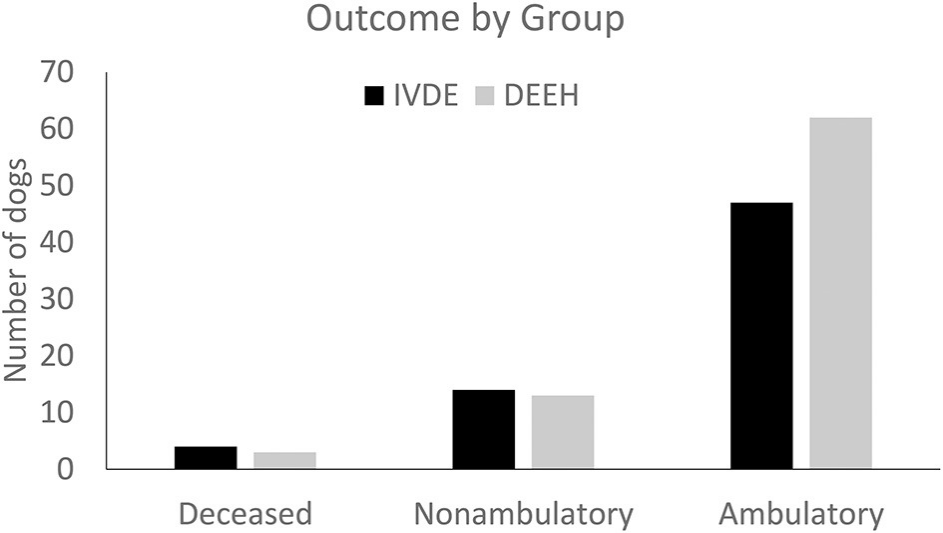
Patient outcomes at the time of follow-up for IVDE and DEEH.

We found no differences in successful outcomes between dogs with IVDE or DEEH compression at any level of neurological severity (i.e., when comparing dogs with similar Modified Frankel Scores; *P* = 0.77). Therefore, we examined differences in overall successful outcomes between the two groups, ignoring the presenting degree of severity. Similar proportions of dogs achieved successful outcomes in each group (*P* = 0.66): 62 IVDE dogs and 47 DEEH compression dogs achieved functional ambulation (success), while 13 IVDE dogs and 14 DEEH dogs failed to ambulate (failure) (Figure 7). When examined by presenting Modified Frankel Scores, the probability of a successful outcome increased with decreasing severity (*P* < 0.0001).

## Discussion

We found that dogs demonstrating myelopathic signs secondary to DEEH compression have no difference in outcome when compared to a matched population of dogs with IVDE without MRI indicators of inflammatory hemorrhagic compression when treated with routine surgery. Dogs with similar presenting Modified Frankel Scores have similar outcomes, provided the surgeon performs an appropriately extended decompression. The presenting Modified Frankel Score best predicted outcome in both groups of dogs with IVDE.

The association between functional recovery and various metrics has been extensively described for routine IVDE populations (10–14). Individual case series have looked at a variety of parameters looking for any association with outcome for dogs with DEEH compression (4) without a control group. Mateo et al. (8) compared dogs with DEEH compression on imaging to non-DEEH compression cases but did not control for neurological severity when selecting their surgical cases. Our study is the first to compare IVDE with DEEH compression to a control group of dogs with IVDE without DEEH compression with similar presenting Modified Frankel Scores. Our study found no overall difference in outcome after surgery between these two groups. Both groups of dogs had similar distributions of presenting Modified Frankel Scores, so we could appropriately compare overall success rates.

Compared to Woelfel et al. (4), our DEEH compression group had a smaller body weight. There was a significant difference between our control group's body weight and the DEEH compression group. Our DEEH group was heavier than the control group but somewhat lower in mean body weight than affected dogs represented in the Woelfel et al. (4) study. This discrepancy reflects differing inclusion criteria between these two studies. Our DEEH group included dogs of all body weights, and any neurological status at time of admission to the hospital. The population with DEEH in Woelfel et al. (4) was restricted to patients >10 kg in body weight, and with a neurological status of paraplegia. In our study successful surgical outcome was not correlated with body weight.

We found that the compression to decompression ratio did not differ between the two groups of dogs, even though dogs with DEEH compression had larger regions of compression and, consequently, larger decompressive regions. Therefore, surgeons performing decompression of dogs with DEEH compression can expect similar outcomes to dogs with non-hemorrhagic IVDE, provided they appropriately decompress sufficient sites. Larger surgical decompression has been associated with a more favorable outcome, however previously this was in relation to MRI indicators of potential spinal cord swelling (4). The present study assessed the vertebral canal for compression to simulate surgical decisions of which vertebral levels will be opened.

We used the presenting Modified Frankel Score to stratify dogs into different severities. The Modified Frankel Score is a simple evaluation tool; a more complex scoring system, such as the Texas Spinal Cord Injury scale, or a 14-point motor score, might more accurately evaluate success (15). While the TSCI scale might have allowed for a more granular breakdown of the different groups to better interrogate any relationship between outcomes in these groups, the Modified Frankel Score has been validated and shown to be accurate for retrospective studies (10, 12, 16).

There are few reports of how large a decompressive hemilaminectomy can be before surgeon concerns about the instability of the spine might preclude further surgical decompression. The biomechanics of three contiguous ipsilateral hemilaminectomy sites or two continuous bilateral sites exhibited no major changes in stability in a cadaver study (17). Suggested models of stability based on spinal fractures suggest that if two out of three compartments are intact, that individual location is not unstable (18). The approach for routine hemilaminectomies involves disruption of only the dorsal compartment; therefore, a single site hemilaminectomy may not be inherently destabilizing. Peer-reviewed studies have not evaluated how changes to the dorsal compartment of a large number of consecutive vertebrae affect vertebral column stability. However, given the available studies, coupled with the results of the present study, surgeons should consider an extensive surgical decompression to give the best chance of functional recovery.

Due to the retrospective nature of this study, the follow-up time frame for these cases was varied and on average, the DEEH cases were re-evaluated later after surgery (median 6 weeks) than the control group (median 3 weeks). The mean time to ambulation in dogs with IVDE has been previously reported between 2 and 7 weeks following surgery but has not been specifically reported in DEEH cases (19). It is possible that with a longer time frame of follow-up that some dogs given a success grade of 1 (alive and non-ambulatory) in this study may have continued to improve to regain ambulation.

Our study relied on imaging parameters to assign cases to the DEEH compression group, or non-DEEH compression control group. This is a limitation in that material was not collected and submitted for histopathology, and the MRI findings were not confirmed through biopsy of material from both groups. (7) indicated that hemorrhage and inflammatory infiltrate can be found on histopathology for most cases of IVDE.

Our DEEH compression population had significantly larger decompressive laminectomies, which is likely guided by the bias of the attending neurosurgeon for decompressing over the entire area of compression noted on MRI. Therefore, we cannot evaluate whether less extensive decompressive surgery might have resulted in similar outcomes. There could be a minimum threshold of compression to decompression ratio that allows for the smallest possible decompression with extensive compressive lesions. The DEEH compression population in this study did not have enough variation in compression to decompression ratio to investigate this.

In conclusion, dogs with disk extrusions with or without DEEH compression on their MRIs are expected to have a successful outcome when their compression is addressed surgically with appropriate decompression. Presenting Modified Frankel Score, rather than the extent of extradural compression visualized on MRI or presence of hemorrhage, predicts functional outcome.

## Data Availability Statement

The raw data supporting the conclusions of this article will be made available by the authors, without undue reservation.

## Author Contributions

PL and JW designed and coordinated the study with the support of PJ, IP, NA, and MR. PL, JW, PJ, and NA provided all the necessary information from the clinical databases of the participating referral hospital. PL accessed and centralized all the clinical information. MR performed all the statistical analysis. PL processed the results and wrote the manuscript under the supervision and coordination of JW. All authors read refined and approved the final manuscript.

## Conflict of Interest

The authors declare that the research was conducted in the absence of any commercial or financial relationships that could be construed as a potential conflict of interest.

## Publisher's Note

All claims expressed in this article are solely those of the authors and do not necessarily represent those of their affiliated organizations, or those of the publisher, the editors and the reviewers. Any product that may be evaluated in this article, or claim that may be made by its manufacturer, is not guaranteed or endorsed by the publisher.
